# Structural and Functional Implications of the Interaction between Macrolide Antibiotics and Bile Acids

**DOI:** 10.1002/chem.201406413

**Published:** 2015-02-05

**Authors:** Simon Glanzer, Sergio A Pulido, Sarah Tutz, Gabriel E Wagner, Manfred Kriechbaum, Nina Gubensäk, Jovana Trifunovic, Markus Dorn, Walter M F Fabian, Predrag Novak, Joachim Reidl, Klaus Zangger

**Affiliations:** [a] Institute of ChemistryUniversity of Graz (Austria); [b] Institute of Molecular BiosciencesUniversity of Graz (Austria); [c] Institute of Inorganic ChemistryGraz University of Technology (Austria); [d] Dept. of Pharmacology, Medical FacultyUniversity of Novi Sad (Serbia); [e] Institute of NavigationGraz University of Technology (Austria); [f] Department of Chemistry, Faculty of Natural ScienceUniversity of Zagreb (Croatia)

**Keywords:** azithromycin, bile acids, diffusion ordered spectroscopy (DOSY), macrolide antibiotics, micelles, NMR spectroscopy

## Abstract

Macrolide antibiotics, such as azithromycin and erythromycin, are in widespread use for the treatment of bacterial infections. Macrolides are taken up and excreted mainly by bile. Additionally, they have been implicated in biliary system diseases and to modify the excretion of other drugs through bile. Despite mounting evidence for the interplay between macrolide antibiotics and bile acids, the molecular details of this interaction remain unknown. Herein, we show by NMR measurements that macrolides directly bind to bile acid micelles. The topology of this interaction has been determined by solvent paramagnetic relaxation enhancements (solvent PREs). The macrolides were found to be bound close to the surface of the micelle. Increasing hydrophobicity of both the macrolide and the bile acid strengthen this interaction. Both bile acid and macrolide molecules show similar solvent PREs across their whole structures, indicating that there are no preferred orientations of them in the bile micelle aggregates. The binding to bile aggregates does not impede macrolide antibiotics from targeting bacteria. In fact, the toxicity of azithromycin towards enterotoxic *E. coli* (ETEC) is even slightly increased in the presence of bile, as was shown by effective concentration (EC_50_) values.

## Introduction

Macrolide antibiotics have been in clinical use to treat infections of both gram-positive and gram-negative bacteria. They act by down-regulating protein synthesis through inhibition of bacterial ribosomes.[1] About 50 % of the total human drug clearance is accomplished through the biliary pathway.[2] About 60–70 % of macrolide antibiotics are excreted by bile[3] and the remaining part by urine.[4] A tissue disposition study of azithromycin in rabbits has also shown that the highest tissue concentrations of this drug are found in bile.[5] It was hypothesized that biliary macrolide uptake plays a vital role in effectiveness and side effects of macrolide antibiotics in humans. Differences in the bioavailability of drugs and especially macrolides, when taken orally shortly after a meal, have been described.[6] The importance of macrolide antibiotics uptake by bile has also been confirmed by experiments with bile duct cannulated rats, which showed significantly reduced plasma concentrations of roxithromycin.[7] The use of macrolide antibiotics has been reported as the cause for several diseases related to the biliary system. For example, azithromycin- or erythromycin-induced cholestasis is believed to result from hypersensitivity towards these drugs and causes lesions of canalicular membranes, which could lead to vanishing bile-duct syndrome.[8] Macrolide antibiotics have also been shown to influence the biliary excretion of other drugs. Erythromycin inhibits the excretion of ximelagatran and its metabolites.[9] The same macrolide antibiotic has also been found to reverse the bile salt tolerance in *Campylobacter jejuni* and *Campylobacter coli* strains.[10] Despite mounting evidence for the direct interaction between macrolide antibiotics and bile, there is no report about the molecular details of this interaction. Herein, we present the structural and functional details of drug–bile interactions of selected macrolide antibiotics and some of the most common bile acids to explain this difference in bioavailability and effectiveness. We show which features of macrolides are important for interactions with bile. The interaction between macrolide antibiotics and bile acids was investigated by NMR chemical-shift titration, self-diffusion measurements, paramagnetic relaxation enhancements, as well as small-angle X-ray scattering (SAXS), by using bile-acid micelles and also simulated intestinal fluids. The influence of bile acids on the antibiotic activity of macrolides was assessed by activity measurements in a pathogenic *E. coli* strain (*enterotoxic E. coli* ETEC).

## Results and Discussion

**Figure 1:**
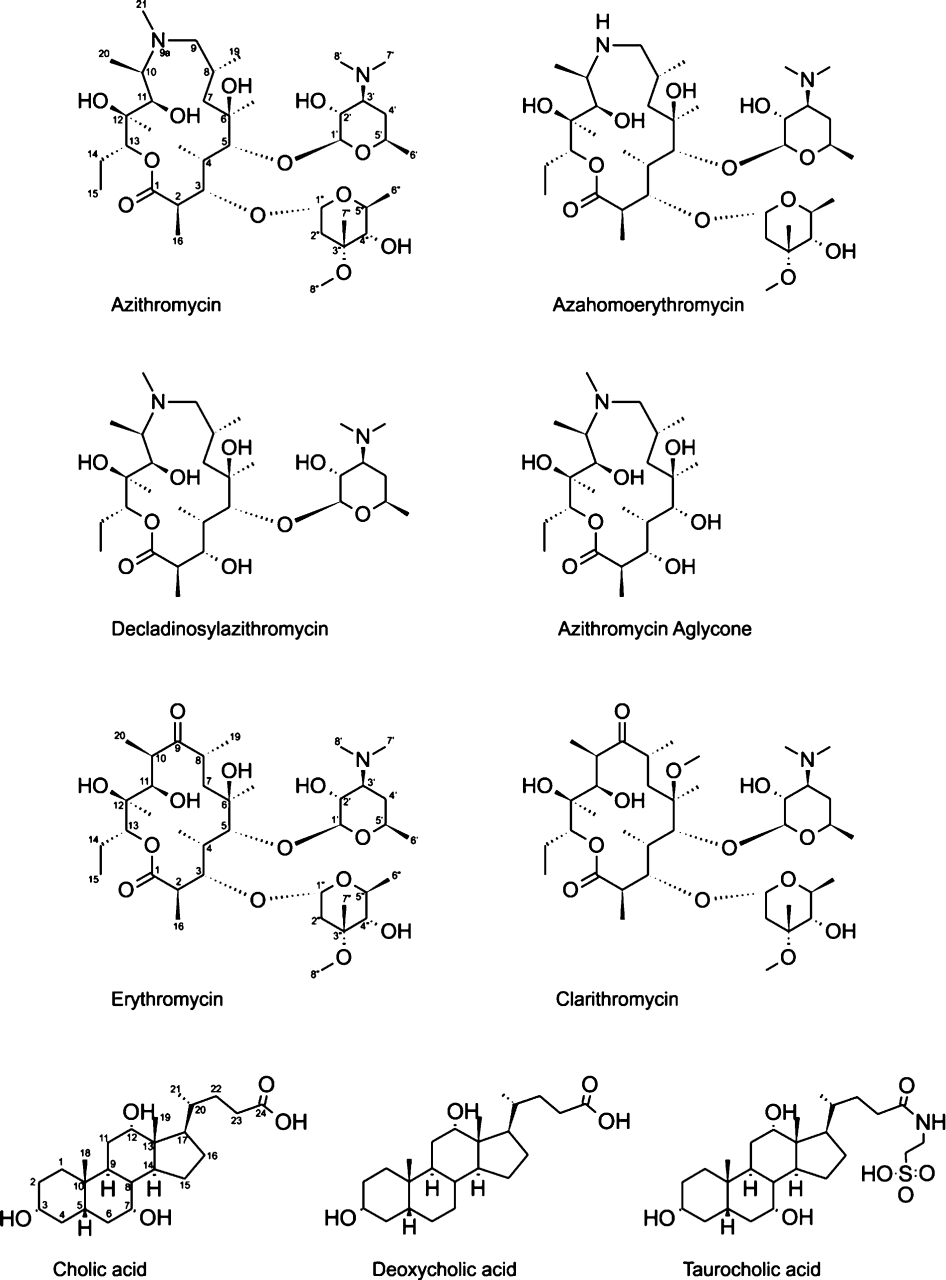
Structures and numbering schemes for the macrolides and bile acids used.

For this study, a series of representative macrolides were used. In particular, the antibiotics azithromycin, erythromycin, and clarithromycin, but also the macrolides azahomoerythromycin, decladinosylazithromycin, and azithromycin aglycone, which show no antimicrobial activity. The chemical structures of all commonly used macrolide antibiotics are very similar. They consist of a 14- or 15-membered alkylated lactone ring with hydroxyl groups on C3, C5, C6, C11, C12 and a desosamine and decladinose on C3 and C5[11] (Figure 1). Human bile mainly consists of bile acids synthesized from cholesterol, with cholic acid and its deoxo derivatives being the most abundant compounds. In natural bile, the concentration of bile salt is about 40 mm.[12] Structurally, bile acids consist of a hydrophobic nonaromatic four-ring system with hydrophilic hydroxyl groups on one side and a side chain with a carboxyl group on the five-membered ring (Figure 1). In some cases, a glycol or taurine is attached to the carboxyl group. Bile acids are amphiphilic and form primary micelles of 2–10 bile acid molecules at lower (5–15 mm) and vesicles (aggregation number 10–100 aggregates) at higher concentrations.[13] The main physiological functions of bile acids are the solubilization and transport of lipids and interaction with lipid soluble vitamins and drugs[13–15].

### Binding of macrolide antibiotics to bile-acid micelles

**Figure 2:**
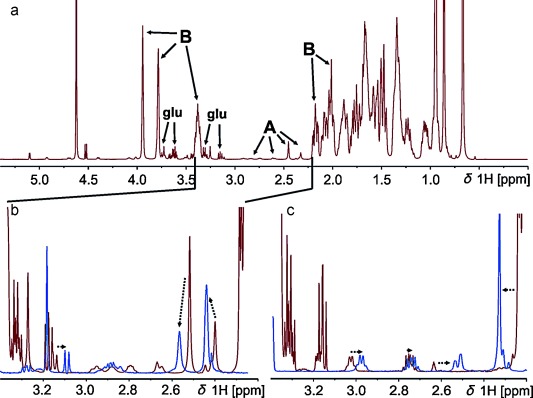
a) 1D ^1^H NMR spectrum of azithromycin dihydrate in a 50 mm sodium cholate solution. All intense peaks belong to bile (annotated with B), whereas only a small gap from 2.2–3.4 ppm is open for analysis of the small macrolide signals (labeled A). b) Zoom into the region between 2.2–3.4 from the spectrum above (red) and, for comparison, in blue: free azithromycin in HBSS. The dotted arrows indicate shifts from free to bile-bound azithromycin. c) Zoom in the same region, but of azithromycin aglycone in the absence (blue) and presence (red) of 100 mm sodium cholate. Signals of glucose (from Hank’s buffer) are labeled glu.

Information about the interaction between macrolide antibiotics and bile-acid micelles can be obtained by NMR titration measurements. The recorded proton (^1^H) NMR spectra are dominated by the huge bile-acid signals, which is typically used at concentrations between 20 and 50 mm. In contrast, the macrolides are around 1–2 mm in a saturated solution. Typically, NMR studies of small molecules bound to micelles use deuterated lipids or detergents to significantly reduce the latter signals.[16] Unfortunately, bile acids are not commercially available in deuterated form and any NMR information about the macrolides has to be extracted from “windows” in the bile-acid spectra. To the best of our knowledge, this is the first NMR study of unlabeled ligands bound to non-deuterated micelles. Addition of bile salts leads to significant changes in the ^1^H NMR spectra of all investigated macrolides (Figure 2).

**Figure 3:**
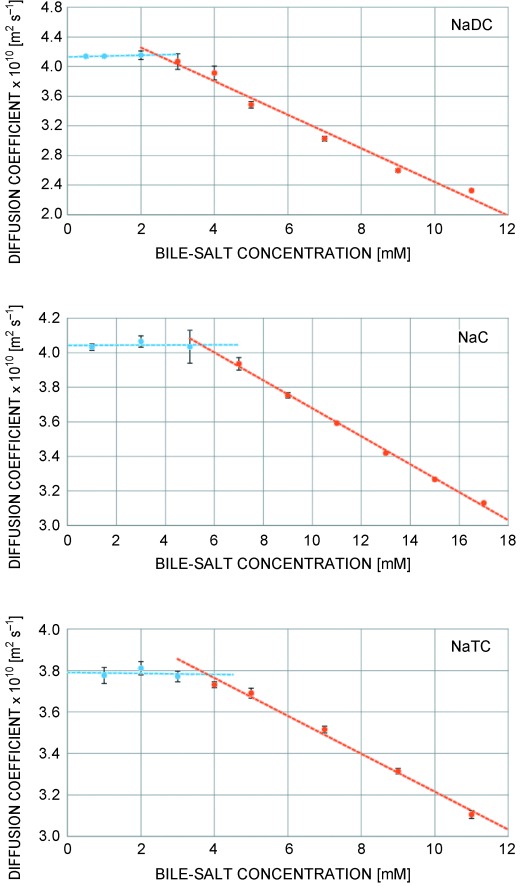
Self-diffusion coefficients of the free bile salts sodium deoxycholate (NaDC), sodium cholate (NaC), and sodium taurocholate (NaTC) in HBSS buffer, measured at 298 K, at varying concentrations above (red) and below (blue) the cmc. The point of intersection represents the cmc.

Binding to the micelles is indicated by differences in the chemical shift[17] of the macrolides and line broadening as a consequence of reduced mobility in the micelle. For the aglycone, some peaks are shifted, but the line shape is less affected, which indicates rather loose binding to micelles. Addition of bile micelles increases the solubility of macrolide antibiotics by a factor of approximately 2–3. However, to prevent any precipitation in subsequent experiments, we added macrolides from a 100 mm stock solution in DMSO to a final concentration of 1 mm. For a quantitative determination of binding affinities, it is important to study the binding to defined bile aggregates. Therefore, an accurate knowledge of critical micelle concentration (cmc) is needed. Due to large variations of reported cmc of bile salts (8–20 mm for NaC (C=cholate), 3–13 mm for NaDC (DC=deoxycholate), and 18 mm for NaTC (TC=taurocholate)[18]) and to get accurate values in Hank’s balanced salt solution (HBSS) as a solvent, we determined these values by diffusion ordered spectroscopy. For this purpose, the diffusion coefficients of the individual bile salts were determined at different concentrations. At the cmc, micelles begin to form, and the overall diffusion coefficient decreases (Figure 3).

In these experiments, we obtained the following cmc values in HBSS: NaC (4.74±1.16 mm); NaDC (2.52±1.09 mm); and NaTC (3.75±1.66 mm). This cmc pattern can be explained by considering the amphiphilic character of bile. The more the apolar part of the molecule outweighs the polar side, the lower is the solubility of single molecules in an aqueous environment, and therefore also their cmc. The missing OH group on deoxycholate and the attached taurine unit on NaTC reduce the polar part of the bile salt and therefore their cmc. The importance of hydrophobic interactions in the micelle formation is corroborated by cmc values obtained in pure D_2_O solution (see the Supporting Information), which are all higher than in the buffered solution. Ions in solution destabilize less polar groups on the bile acids and lead to their more favorable protection through micelle formation. Bile-acid micelle dimensions depend on their concentration. Larger aggregates are typically found at higher concentrations.[19,20] To ensure that all binding experiments are carried out in a solution containing micelles of a defined size, we used bile salt concentrations of 50 mm for NaC and NaTC and 25 mm for NaDC. Due to their limited solubility, the macrolide antibiotics were added from a 100 mm stock solution in DMSO to a final concentration of 1 mm, which is slightly below the solubility of azithromycin and clarithromycin in water. The macrolide solubility decreases in the following order: azithromycin aglycone>decladinosylazithromycin>erythromycin≈azahomoerythromycin>azithromycin≈clarithromycin. The solubility is paralleled by their polarity. Starting from erythromycin and azahomoerythromycin with average hydrophobicity, the proton on O6 or N9a is replaced by a methyl group in clarithromycin and azithromycin, respectively, making it more hydrophobic. On the other hand, the two sugars, cladinose and desosamine, are less polar than the macrolide ring. Removing sugar units (decladinosylazithromycin and azithromycin aglycone) increase the polarity. Information about the binding strength between macrolide antibiotic and bile-acid micelles can be obtained from diffusion measurements.[11,21] Due to significant signal overlap in most parts of the proton spectrum, diffusion coefficients of the macrolides could only be obtained in the region between approximately 2.2 and 3.0 ppm. Enhancing the resolution with pure shift DOSY experiments[22] was unsuccessful due to fast transverse relaxation in the large bile-acid micelles.

In this mixture, the experimental diffusion coefficient of the macrolide is a combination of the diffusion coefficient of free antibiotics (*D*_f_) and bound ones (*D*_b_) according to Equation (1):

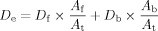
(1)

in which *D*_e_ is the measured diffusion coefficient of the macrolide antibiotic; *A*_b_ is the mole fraction of bound macrolides; *A*_f_ are the free ones; and *A*_t_ is the total amount of macrolides in solution (*A*_t_=*A*_b_+*A*_f_). This equation is also valid for the micelle diffusion coefficient of bile salts. The value for the micellar diffusion can therefore be calculated by the Equation (2):

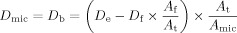
(2)

**Figure 4:**
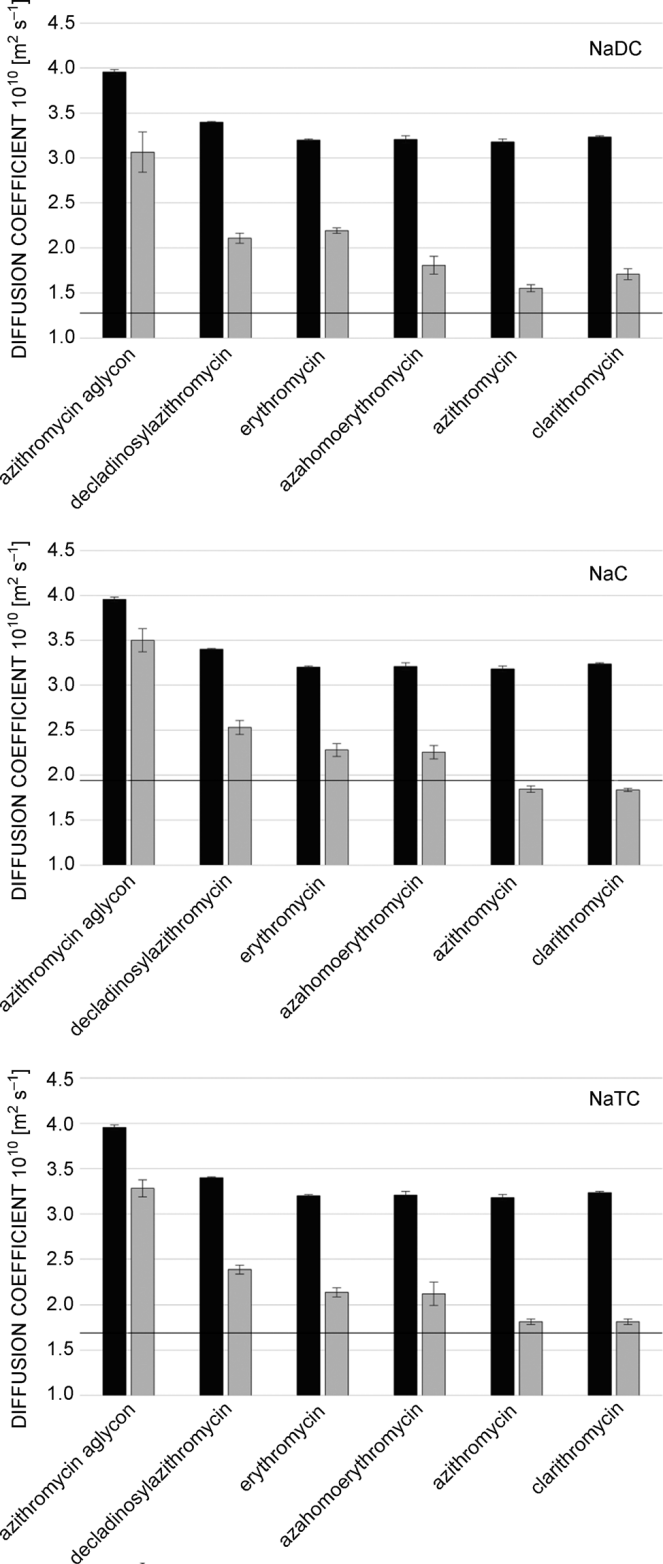
Self-diffusion coefficients of free macrolides (1 mm) in HBSS buffer (black) and in the presence of bile salts (grey). Due to differences in their respective cmc, a concentration of 25 mm was used for sodium deoxycholate (NaDC), while 50 mm were used for sodium cholate (NaC) and sodium taurocholate (NaTC). All values were obtained at 298 K. The horizontal line represents the micellar diffusion *D*_mic_.

Herein, the concentration of free bile molecules (*A*_f_) is the cmc and the diffusion coefficient of the free bile can be obtained from Figure 3. With this information, *D*_mic_ can be calculated using Equation (2) to be 1.90×10^−10^, 1.27×10^−10^, and 1.66×10^−10^ m^2^ s^−1^ for NaC, NaDC and NaTC, respectively. Diffusion measurements of all investigated macrolide antibiotics with the three bile acids show huge differences between free antibiotic and antibiotic+bile mixtures, which confirm the strong interaction and binding of the macrolides to bile micelles (Figure 4). The missing sugars of azithromycin, aglycone and decladinosylazithromycin, reduce their size and raise the diffusion coefficient of free antibiotic. All other macrolides show similar diffusion properties. The addition of all three bile salts has similar effects on the diffusion coefficient of antibiotics. Although the diffusion coefficients of azithromycin aglycone and decladinosylazithromycin are drastically reduced by bile salts, they are less affected than the others. This can be explained by their slightly higher polarity. On the other hand, the apolar macrolides azithromycin and clarithromycin feature a diffusion coefficient very close to the micelles of all three bile salts, which indicates stronger binding. Erythromycin and azahomoerythromycin are also bound to the micelles, but small proportions are free in solution.

Using the diffusion coefficient of the micelles, their hydrodynamic radius can be estimated by using the Stokes–Einstein relation, as was previously reported.[23] This results in radii of 11.5, 17.4, and 13.2 Å for cholate, deoxycholate, and taurocholate micelles, respectively. With the hydrodynamic radii of the free bile salts of 5.4 (NaC), 5.3 (NaDC), and 5.8 Å (NaTC) aggregation numbers (number of bile salt monomers in a single micelle) of 10 (NaC), 35 (NaDC), and 11 (NaTC) result. The estimation of micelle radii and aggregation numbers from diffusion coefficients also depends on the solution viscosity. Due to the low concentration of the used antibiotics (1 mm), these changes are rather minor.[23] However, to get an independent estimate, the micelle size (radius of gyration *R*_g_) of cholate and deoxycholate in the absence and presence of azithromycin was also determined by small-angle X-ray scattering. The *R*_g_ values obtained by this technique are 10.3±0.3 (NaC), 10.1±0.3 (NaC+1 mm azithromycin), 19.8±2.3 (NaDC), and 14.3±2.8 Å (NaDC+1 mm azithromycin). These values are in very good agreement with the hydrodynamic radii obtained by NMR diffusion measurements, and they also confirm the slight reduction in micelle size of deoxycholate upon macrolide binding (Figure 4). The radius of gyration, as well as the diffusion behavior obtained for micelles of sodium cholate, is within the experimental uncertainty in the absence and presence of macrolides. For these micelles, a bile salt concentration of 50 mm is used, but only 1 mm of macrolide is added. For deoxycholate, a concentration of 25 mm has been employed due to its lower cmc, and herein, clearly the binding of macrolides leads to a slight reduction of the micelle size, which however is still close to the standard deviation. The observed radii and diffusion coefficients of sodium deoxycholate in the presence of antibiotics correspond to a reduction of its aggregation number from approximately 35 to approximately 25. A reduction in aggregation number of NaDC micelles by macrolide binding could explain the increase in polarity. NaDC is the most hydrophobic bile acid used in this study, and the relatively high number of hydroxyl groups on macrolides increases the overall polarity, and therefore slightly reduces the micelle size. In general, the higher aggregation number and lower cmc of NaDC compared with NaC and NaTC is somewhat surprising and might be related to a different shape of these micelles. Anyway, the binding behavior of macrolide antibiotics is basically not influenced by this different aggregation behavior, as was seen by the rather similar trends of the diffusion coefficient in different bile micelles.

**Figure 5:**
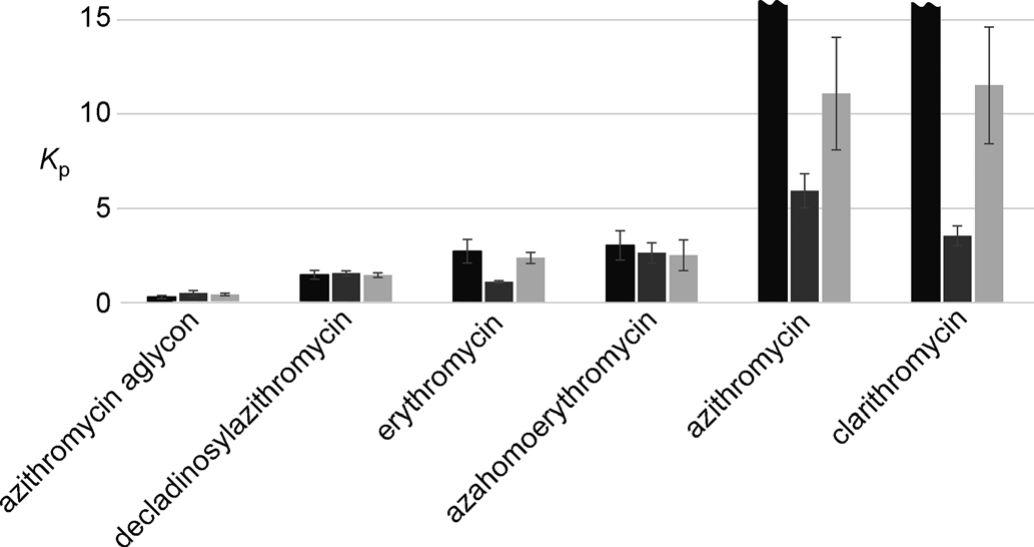
Mole fraction partition coefficient (*K*_p_), which is the ratio between bound and free ligand, is shown for all investigated macrolides (total concentration 1 mm) for cholate (black), deoxycholate (dark grey), and taurocholate (light grey). Due to differences in their respective cmc, a concentration of 25 mm was used for sodium deoxycholate (NaDC), whereas 50 mm were used for sodium cholate (NaC) and sodium taurocholate (NaTC). All values were obtained at 298 K. Mole fraction partition coefficients above approximately 15 are unreliable due to huge standard deviations and are indicated by the wave-shaped ends.

Quantitative information about the strength of the interaction between micelles and a ligand is often expressed by the mole fraction partition coefficient *K*_p_, which is the ratio of bound to free-ligand molecules. It can be obtained through diffusion measurements of free and bound ligand (macrolides) and bile micelles, and is given by Equation (3):[11]

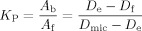
(3)

in which *D*_e_, *D*_f_, and *D*_mic_ refer to the diffusion coefficients of antibiotic in bile salt, free antibiotic, and bile-salt micelles, respectively. For very tightly bound ligands, the diffusion coefficient of the bound form (*D*_e_) is close to the one of the micelle (*D*_mic_), which makes the estimation of *K*_p_ values prone to errors. Therefore, only upper limits can be given. This is the case for azithromycin and clarithromycin in the presence of cholate and deoxycholate. Using the upper limit within the standard deviation of *D*_e_ and the lower limit for *D*_f_, it is possible to calculate a minimum value for *K*_p_ (>15 in NaC) for these two antibiotics (Figure 5 and Table 1).

**Table 1:** Mole fraction partition coefficients in NaC 50 mm, NaDC 25 mm, and NaTC 50 mm.

	Azithromycinaglycone			Decladinosyl-azithromycin			Erythromycin			Azahomoerythromycin			Azithromycin			Clarithromycin		
NaC	0.29	+/−	0.09	1.47	+/−	0.24	2.73	+/−	0.63	3.03	+/−	0.78		–[*]			–[*]	
NaDC	0.50	+/−	0.14	1.55	+/−	0.13	1.10	+/−	0.05	2.64	+/−	0.53	5.94	+/−	0.91	3.55	+/−	0.53
NaTC	0.42	+/−	0.07	1.46	+/−	0.12	2.73	+/−	0.30	2.51	+/−	0.81	11.1	+/−	3.0	11.5	+/−	3.1

[*] *K*_p_ values above approximately 15 are unreliable due to huge standard deviations.

Due to the relatively high polarity of azithromycin aglycone, it shows the lowest ratio of bound to free molecules of all macrolides tested. The additional sugar unit of decladinosylazithromycin raises the *K*_p_ to approximately two. Erythromycin and azahomoerythromycin bind tightly to the bile-salt micelles; only 20–30 % remain as free molecules. Basically everything of clarithromycin and azithromycin is bound in the NaC and NaTC micelles, giving high *K*_p_ values over ten. This can be explained by their highest hydrophobicity, which makes dissolution in the aqueous phase energetically disfavored. Binding to bile is important for both transport and excretion of macrolide antibiotics. Strong binding leads to fast transport of the antibiotics to their target site, but also faster excretion. More hydrophobic macrolides should therefore be more suitable when fast action is needed, whereas more polar ones might prove to be useful for long-term treatment, when excretion should be as slow as possible.

### Binding in intestinal body fluids

**Figure 6:**
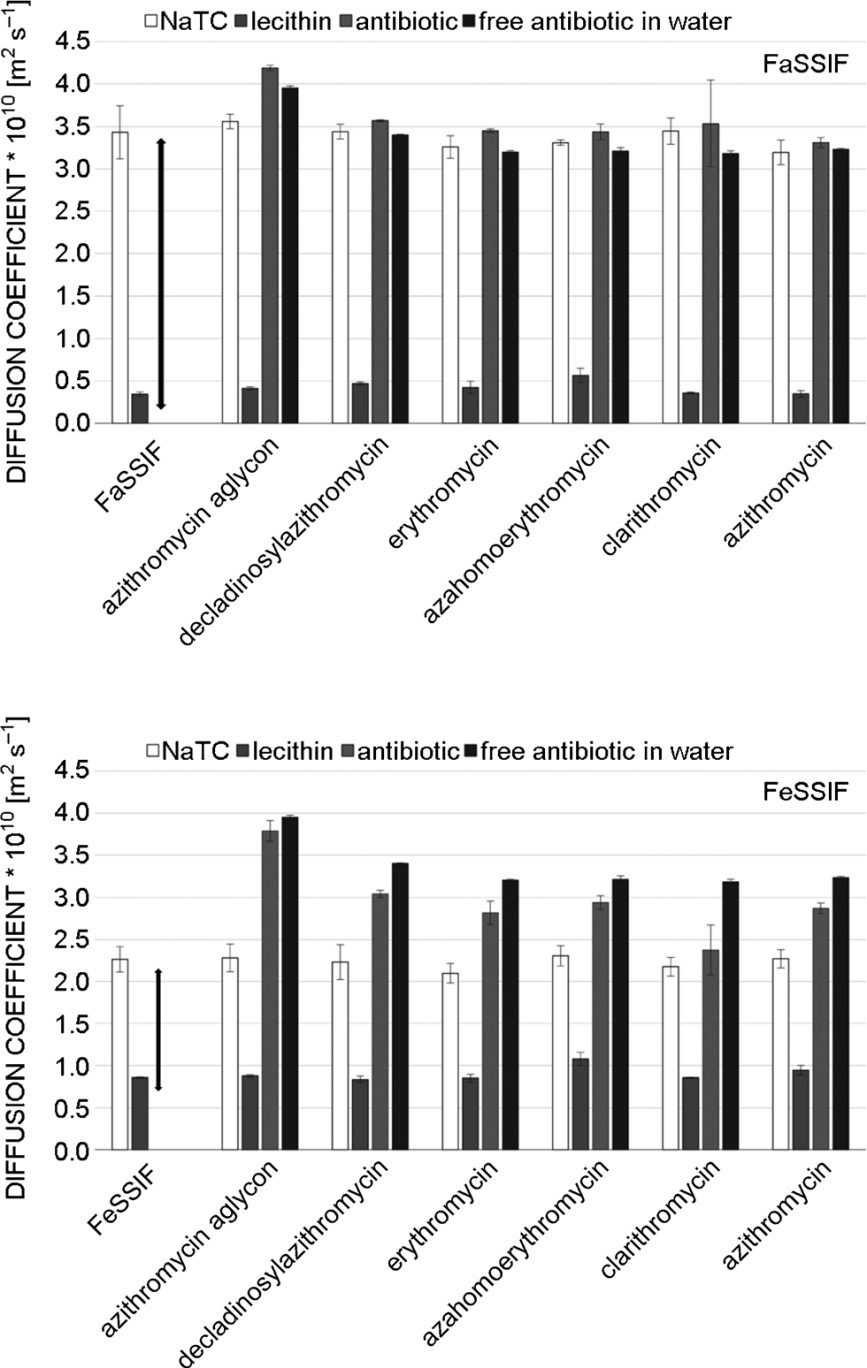
Two investigated bile mimetics: FeSSIF contains NaTC above the cmc of mixed micelles and FaSSIF below. Hence, NaTC and lecithin show more similar diffusion. The mixed micelles bind the macrolides and lower their diffusion coefficient.

To investigate the binding of macrolides to bile-acid aggregates under more physiological conditions, fasted- and fed-state simulated intestinal fluids (FaSSIF and FeSSIF, respectively) were used. The difference between these two solutions is based on a 1+4 dilution of FeSSIF with HBSS to obtain FaSSIF. It is suggested in the literature that lecithin and NaTC form mixed micelles with a cmc between 5–12 mm.[15] Therefore, in FaSSIF, no mixed micelles are formed, resulting in a high diffusion coefficients of monomeric bile acid (Figure 6). In contrast, the diffusion of lecithin is much slower, indicating the formation of large aggregates of this lipid. The fast diffusion of the macrolides in this solution is close to that of the same compounds in an aqueous environment, indicative that they do not bind to lecithin micelles. On the other hand, in FeSSIF, mixed micelles are formed, and can uptake the macrolides and transport them through the body. The only slight reduction of the macrolide diffusion coefficient is probably a combination of the presence of highly hydrophobic lecithin in these mixed micelles and the rather low molar ratio of the mixed micelles to macrolide. As was mentioned above, a cmc around 5–12 mm has been reported for FeSSIF. The concentration of NaTC is 15 mm, so 3–10 mm are found in micelles. The aggregation number of pure NaTC micelles is approximately eleven. If it is similar in mixed micelles, this would result in a mixed micelle concentration between 0.3 and 1 mm. It should also be mentioned that in vivo, a concentration of 1 mm macrolide would be extremely high. At lower concentrations, the ratio of macrolide bound is probably much higher. It is surprising that the macrolides do not interact with the lecithin aggregates, which would lead to lower *D* values of them in FaSSIF medium. Binding to bile acid and mixed micelles clearly does not only occur through hydrophobic interactions in the micelle, but has to involve some other interactions as well. Macrolides contain a number of polar groups, which clearly prevent them from binding to highly hydrophobic aggregates. To get a better idea about the exact mode of binding, it is important to know the topology of the macrolides in the micelles.

### Orientation and localization of macrolides in bile micelles

**Figure 7:**
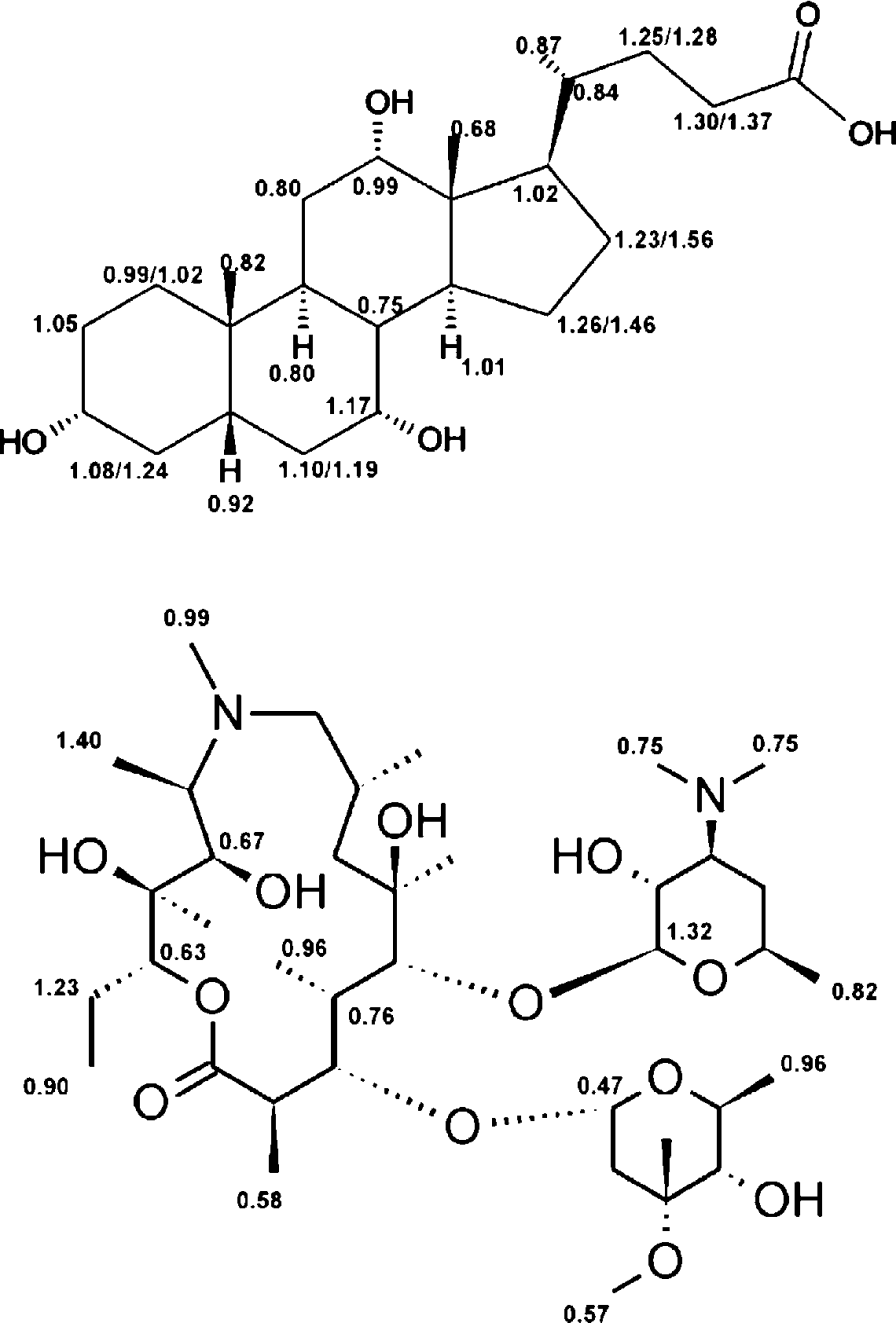
Solvent PREs [mm^−1^s^−1^] of cholate and azithromycin.

To investigate the localization and mode of binding of macrolide antibiotics to bile-acid micelles, we used solvent paramagnetic relaxation enhancements (PREs).[24] Addition of an inert, highly soluble, paramagnetic compound to the solvent leads to a “paramagnetic solvent”.[25,26] A paramagnetic center causes distance-dependent relaxation rate enhancements. When the whole solvent is paramagnetic, this gives information about the immersion depth of an atom in a macromolecular assembly. For micelles, these solvent PREs decay with approximately 1/*d*^3^, in which *d* is the insertion depth.[25] Therefore, atoms close to the surface of the micelle are more affected than ones, which are further inside the micelle. Due to the dominating bile-salt peaks in the spectra, quantitative solvent PREs could be obtained only for a handful of isolated macrolide peaks in ^1^H–^13^C HSQC spectra. Due to their favorable long-term stability, we determined solvent PREs in sodium cholate micelles. The inert paramagnetic agent gadolinium–diethylenetriamine pentaacetic acid bismethylamide (Gd(DTPA–BMA)) was added up to 2 mm, and solvent PREs obtained by fitting the longitudinal relaxation rates as a function of the gadolinium concentration. We have shown previously that Gd(DTPA–BMA) is very inert, not only towards peptides and proteins, but also hydrophobic micelles.[27] Freely soluble molecules show solvent PREs on the order of 3–10 s^−1^ mm^−1^, ones near the surface of a micelle are typically about 1 s^−1^ mm^−1^ and they decrease down to approximately 0.3 s^−1^ mm^−1^ for protons in the middle of a typical small micelle with a diameter of 20 Å.[25–27] The solvent PREs of azithromycin in NaC micelles are shown for both the bile acid and the macrolide in Figure 7.

In general, all cholate solvent PREs are relatively high, indicative of high solvent accessibility. This can be explained by free bile molecules in the solution with concentrations around the cmc (4 mm). Experimental PREs are an average between the very high, typically about 5–10 s^−1^ mm^−1^ solvent PREs of monomeric bile-acid molecules and the ones in the micelle. For sodium cholate, no clear trend of PREs is found on the structure. All values are close to 1 mm^−1^ s^−1^, which indicates that in the micelle, the individual bile-acid molecules are arranged in a more or less random fashion. There is no part of the molecule that is on average closer to the surface than others. This is in clear contrast to lipid micelles, in which the hydrophobic chains are definitely oriented towards the center of the micelle and the hydrophobic parts on the surface, as was evidenced nicely by solvent PREs.[25,28] Bile acids do not show a clear separation between hydrophobic and polar regions and are therefore randomly oriented in the micelle. The structure of bile-acid micelles is believed to be elongated with hydrophobic parts pointing towards the center of the aggregate.[20] Our experimental solvent PREs do not provide any evidence for a preferred orientation of some part of the structure towards the surface. However, structural averaging of bile aggregates has been described before[20] and is certainly one reason for the rather uniform solvent PREs. Additionally, bile acids show a rather “shallow” separation between hydrophobic and more polar characteristics, which should also contribute to small differences in PREs. For azithromycin, the solvent PREs are in a similar range and also do not show any clear preference for higher or lower values on any part of the structure. Similar to bile acids, macrolides also do not contain any clearly hydrophobic part and are therefore also randomly oriented in the micelle. Their relatively high solvent PREs together with the high mole fraction partition coefficient can only be explained with an average localization close to the surface of the micelle. In addition, the structure of azithromycin contains several structural degrees of freedom. In particular, the attached sugar units can be easily rotated, which also contributes to an averaging of their relative membrane position and thus solvent PREs. In previous studies, it was found that in dodecylphosphocholine (DPC) micelles[11] and bicelles consisting of dimyristoylphosphatidylcholine (DMPC) and dihexanoylphosphatidylcholine (DHPC) vesicles,[29] respectively, macrolide antibiotics are oriented with their amino groups closer to the surface of the membrane mimetic than the rest of the molecule. However, the random orientation of azithromycin in buffered bile micelles is in parallel with the random orientation of sodium cholate. Because both molecules do not contain a clear separation of polar and hydrophobic structural features, they are not restricted in their orientation in the micelle. Therefore, the binding and excretion of macrolide antibiotics through the biliary system is probably governed by the same factors that promote binding of macrolides to membranes, that is, in essence the hydrophobicity. This also indicates that the drugs with the highest *K*_p_ values with bile salts (azithromycin and clarithromycin) are excreted most efficiently through bile. The presented determination of binding to bile micelles in vitro might prove useful as a general easily accessible test system to investigate the potential of a drug for excretion by bile or the use of micelles for drug delivery.[30] Because all tested macrolides bind to bile-acid micelles, the question arises whether this interaction prevents these drugs from inhibiting protein synthesis on the bacterial ribosomes, or at least lowers their activity. However, the binding to micelles with *K*_p_ values of approximately 10–15 is still a rather weak interaction, because approximately 7–10 % of the antibiotics are free in solution. The dissociation constants of macrolide antibiotics bound to ribosomes are in the low nanomolar range, which is much stronger. To investigate the impact of macrolide–bile interactions on the antimicrobial activity, we carried out in-cell experiments on enterotoxic bacteria.

### In-cell experiments

**Figure 8:**
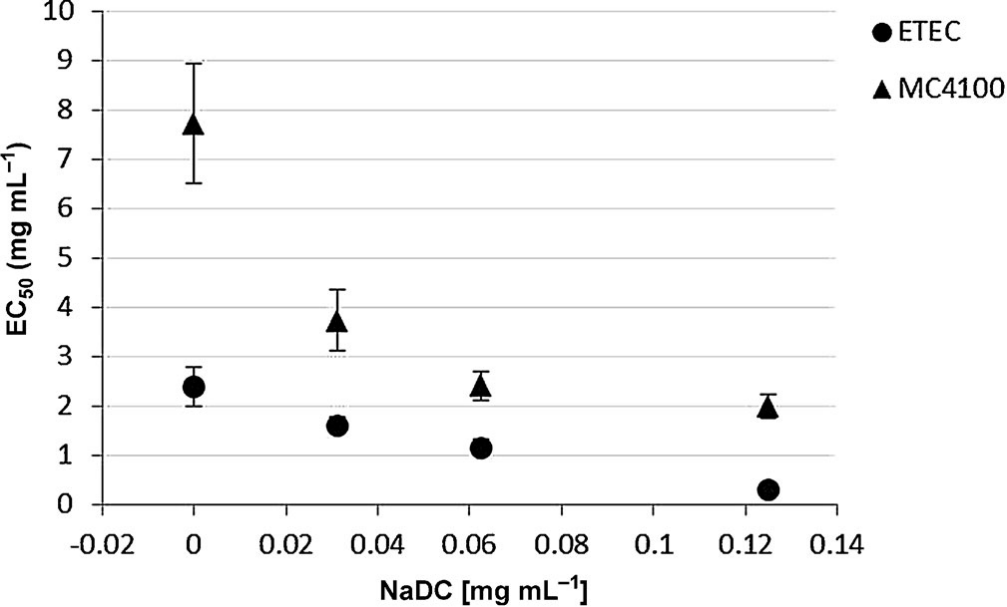
EC_50_ values of azithromycin as a function of NaDC concentration on enterotoxic *E. coli* ETEC H10407 and MC4100-Ery *E. coli* cells. •=ETEC; ▴=MC4100.

As was reported before,[31] different *E. coli* strains exert variable susceptibilities to macrolide antibiotics as a result of several resistance mechanisms widespread between *Enterobacteriaceae*. Herein, we also found differences in the effective concentration 50 (EC_50_) of azithromycin for the tested *E. coli* strains (ETEC H10407 and MC4100) (Figure 8). The EC_50_ is defined as the concentration of antibiotic that reduces the cell growing to the halfway between the control (absence of antibiotic) and the maximum observable growth inhibition. As shown in Figure 8, the presence of NaDC has an effect on the EC_50_ values of azithromycin in both strains. Considering that the concentrations of NaDC (see also Figure S4 in the Supporting Information) used are below the toxic concentrations for the cell, this shows that the toxicity of azithromycin and bile act synergistically.[32] The antimicrobial activity of azithromycin is slightly enhanced by the presence of bile. Whether such collaborative effect involves physical interactions between the compounds or more complex physiological effects is a matter beyond the objective of this experiment.

## Conclusion

We have shown that all macrolides tested (antibiotics, but also non-active ones) bind to different bile acids with varying affinities. Strongest binding has been observed between the most hydrophobic macrolides, azithromycin and clarithromycin, to cholate and deoxycholate micelles, respectively.

To reduce binding to bile and therefore the excretion rate, the use of less hydrophobic antibiotics is advisable, especially if macrolides have to be used for long-term treatment. On the other hand, stronger binding to bile acids would also lead to faster transport through the body and could thus be advantageous when antibiotic activity is needed quickly at the infection site. The macrolides are bound rather close to the surface of bile micelles, with no defined preferred orientation. Macrolides also interact with mixed micelles of taurocholate- lecithin, but no binding is evident to lecithin liposomes. The interaction with bile does not impede the antimicrobial activity of azithromycin in cells, but actually enhances the toxicity of bile and vice versa.

## Experimental Section

Azithromycin, Hank’s balanced salt solution (HBSS), lecithin (ca. 60 %), and HEPES (>99.5 %), and all bile salts were purchased from Sigma–Aldrich (St. Louis, MO, USA) in the highest purity available. All NMR samples were prepared in HBSS (consisting of NaCl 8 g L^−1^, KCl 0.4 g L^−1^, KH_2_PO_4_ 60 mg L^−1^, glucose 1 g L^−1^, phenol red 10 mg L^−1^, Na_2_HPO_4_ 48 mg L^−1^, MgSO_4_ 98 mg L^−1^, CaCl_2_ 140 mg L^−1^, and NaHCO_3_ 0.35 g L^−1^), which was lyophilized and re-dissolved twice in 100 % D_2_O from Eurisotop (99.90 % purity). Simulated intestinal fluids were prepared for the fasted state (FaSSIF: HBSS solvent, 19.45 mm glucose, 10 mm HEPES, 3 mm NaTC, 0.75 mm lecithin) and the fed state (FeSSIF: HBSS solvent, 19.45 mm glucose, 10 mm HEPES, 15 mm NaTC, 3.75 mm lecithin).[15,33].

### NMR spectroscopy

For the interaction studies by NMR spectroscopy, rather higher concentrations of bile acids (25–50 mm) were used to be well above the critical micelle concentration. Due to the limited solubility of macrolides in aqueous medium, they were added from a 100 mm stock solution in DMSO to give a final concentration of 1 mm. Experimentally, we examined azithromycin aglycone, decladinosylazithromycin, erythromycin A, azahomoerythromycin, azithromycin dehydrate, and clarithromycin. The investigated sodium bile salts were cholate (NaC), deoxycholate (NaDC) and taurocholate (NaTC). FeSSIF and FaSSIF were prepared as described (HBSS must be lyophilized and mixed with D_2_O).[15] To obtain self-diffusion coefficients, we used two-dimensional diffusion ordered spectroscopy (DOSY).[34] The employed pulse sequence was a bipolar pulse pair longitudinal eddy current delay (BPP-LED) sequence,[35] with the calibration parameters: 100 ms diffusion time and a 2 ms gradient pulse. In contrast to conventional stimulated echo experiments, bipolar pulse pairs were used to reduce eddy current distortions and during an additional delay, magnetization was stored in the *z* (longitudinal) direction, allowing the eddy current to decay. Paramagnetic relaxation enhancement (PRE) measurements were done on azithromycin in NaDC. Series of six saturation recovery 2D ^1^H–^13^C HSQC spectra at five different Gd(DTPA–BMA) concentrations (0, 0.5, 1, 1.5, and 2 mm) were recorded.[25] The PRE values were obtained by fitting the relaxation rates as a function of gadolinium concentration. The PRE measurements were carried out at 298 K on a Bruker Avance III 700 MHz NMR spectrometer by using a 5 mm cryogenically cooled TCI probe with *z*-axis gradients. All other NMR experiments were carried out at 298 K on a 500 MHz Bruker Avance III NMR spectrometer, equipped with a 5 mm TXI probe with *z*-axis gradient.

### Small-angle X-ray scattering

For small-angle X-ray scattering (SAXS) experiments, we used a high-flux SAXSess camera (Anton Paar, Graz, Austria) connected to a Debye flex 3003 X-ray generator (GE-Electric, Germany), operating at 40 kV and 50 mA with a sealed-tube Cu anode. The Goebel mirror focused and Kratky slit collimated X-ray beam was line shaped (17 mm horizontal dimension at the sample) and scattered radiation from the sample (I) measured in the transmission mode was recorded by a one-dimensional MYTHEN-1k microstrip solid-state detector (Dectris, Switzerland), within a magnitude of the scattering vector, *q*, value of 0.1 to 5 nm^−1^. Using Cu_Kα_ radiation of wavelength 0.154 nm and a sample-to-detector distance of 309 mm, this corresponds to a total 2*θ* region of 0.14 to 7°, with 2*θ* being the scattering angle with respect to the incident beam and λ the wavelength of the X-rays.

Samples were filled into a 1 mm (diameter) reusable quartz capillary with sealing screw caps at both ends. All measurements, of the sample and blank, were done in vacuum and at 20°, with an exposure time of 10 min each. The scattering of the blank (buffer or buffer+azithromycin, respectively) were subtracted from the scattering of the sample solutions (micelles or micelles+azithromycin, respectively) after normalizing both spectra to same transmission. From SAXS measurements, the size information of particles on the nm scale can be quickly and simply obtained by evaluating the parameters of the radius of gyration *R*_g_ directly from the scattering curve *I*(*q*).[36,37]. In particular, the following equations can be applied to extract *R*_g_ (and the extrapolated intensity *I*_0_ at *q*=0) from the scattering curve *I*(*q*), Guinier’s law [Eq. (4)]:

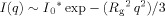
(4)

The data were then analyzed in terms of Guinier’s law,[37] by linearizing the inner part of the scatting curve in a so-called Guinier plot (ln (*I*) vs. *q*^2^), with the linear slope being proportional to the square of the radius of gyration [Eq. (5)]:

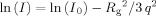
(5)

(linearized Guinier’s law valid at *q* *R*_g_<1)

### In-cell experiments

*Escherichia coli* ETEC H10407 and MC4100[38] strains were taken from frozen stocks and plated into M9 glucose medium. One colony was then inoculated into 20 mL M9 glucose culture flasks and grown overnight under constant shaking. For the determination of the EC_50_ values of azithromycin for each strain, serial dilutions from 0.3 to 200 μg mL^−1^ (ca. 0.5–250 μm) of azithromycin were made in 96 well plates in M9 glucose medium and then inoculated with the respective strain to a final optical density readings OD_600_=0.3. The same setup was used to determine the EC_50_ of the macrolides in the presence of 0.125, 0.0625, and 0.0321 mg mL^−1^ of NaDC (0.3, 0.15, and 0.075 mm, respectively). The plates were then sealed to avoid evaporation and incubated at 37 °C. OD_600_ values were made in a plate reader (Bio-Rad xMark Microplate Absorbance Spectrophotometer) after 20 h to monitor the cell growing. All experiments were performed in triplicate.
