# Supplementary material for: Structural and Functional Implications of the Interaction between Macrolide Antibiotics and Bile Acids
Source: Chemistry. 2015 Feb 5;21(11):4350–8. doi: 10.1002/chem.201406413 (PMC4471570; doi:10.1002/chem.201406413)
Supplement: Supplementary file 1 [file chem0021-4350-sd1.pdf]

CHEMISTRY
A European Journal

Supporting Information

© Copyright Wiley-VCH Verlag GmbH & Co. KGaA, 69451 Weinheim, 2015

# Structural and Functional Implications of the Interaction between Macrolide Antibiotics and Bile Acids

Simon Glanzer,[a] Sergio A. Pulido,[a] Sarah Tutz,[b] Gabriel E. Wagner,[a] Manfred Kriechbaum,[c] Nina Gubensäk,[a] Jovana Trifunovic,[d] Markus Dorn,[e] Walter M. F. Fabian,[a] Predrag Novak,[f] Joachim Reidl,[b] and Klaus Zangger*[a]

chem_201406413_sm_miscellaneous_information.pdf

**Supporting Figure 1:**

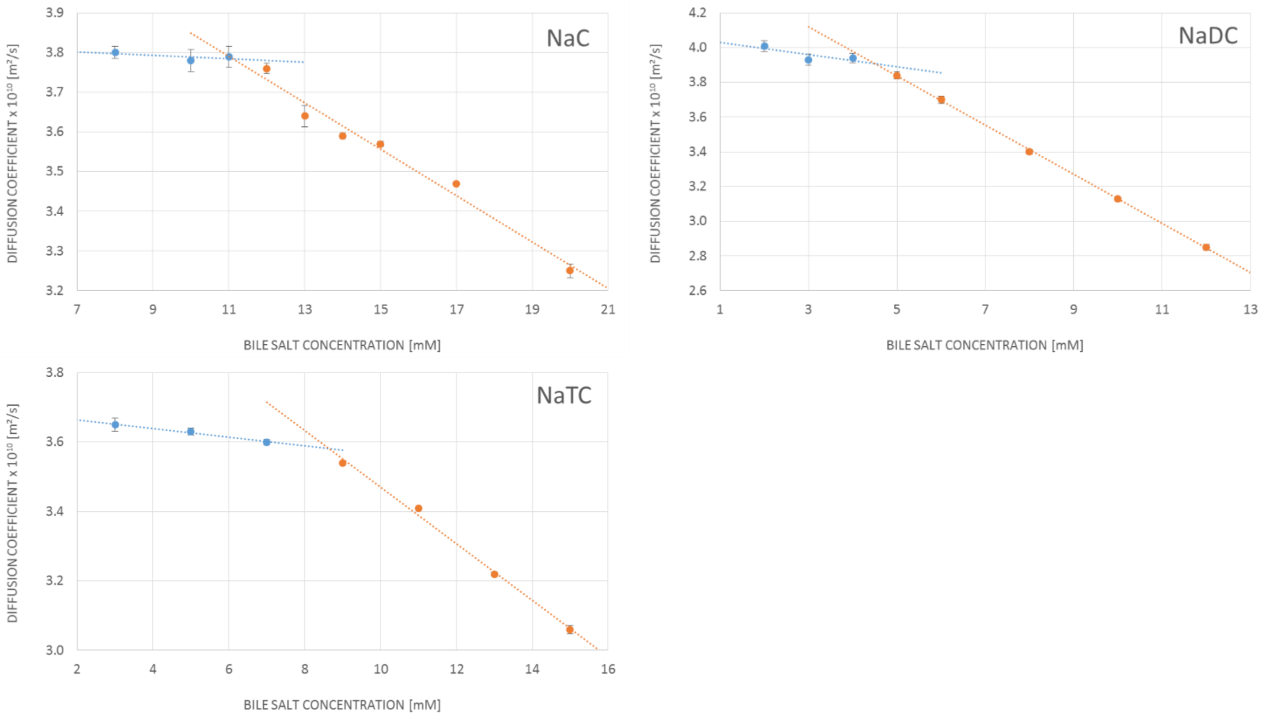


**Supporting Figure 1.** Diffusion values above (red) and below (blue) the cmc in pure $D_2O$. The point of intersection represents the cmc of NaC (~ 11 mM), NaDC (~ 4 mM) and NaTC (~ 9 mM).

**Supporting Figure 2:**

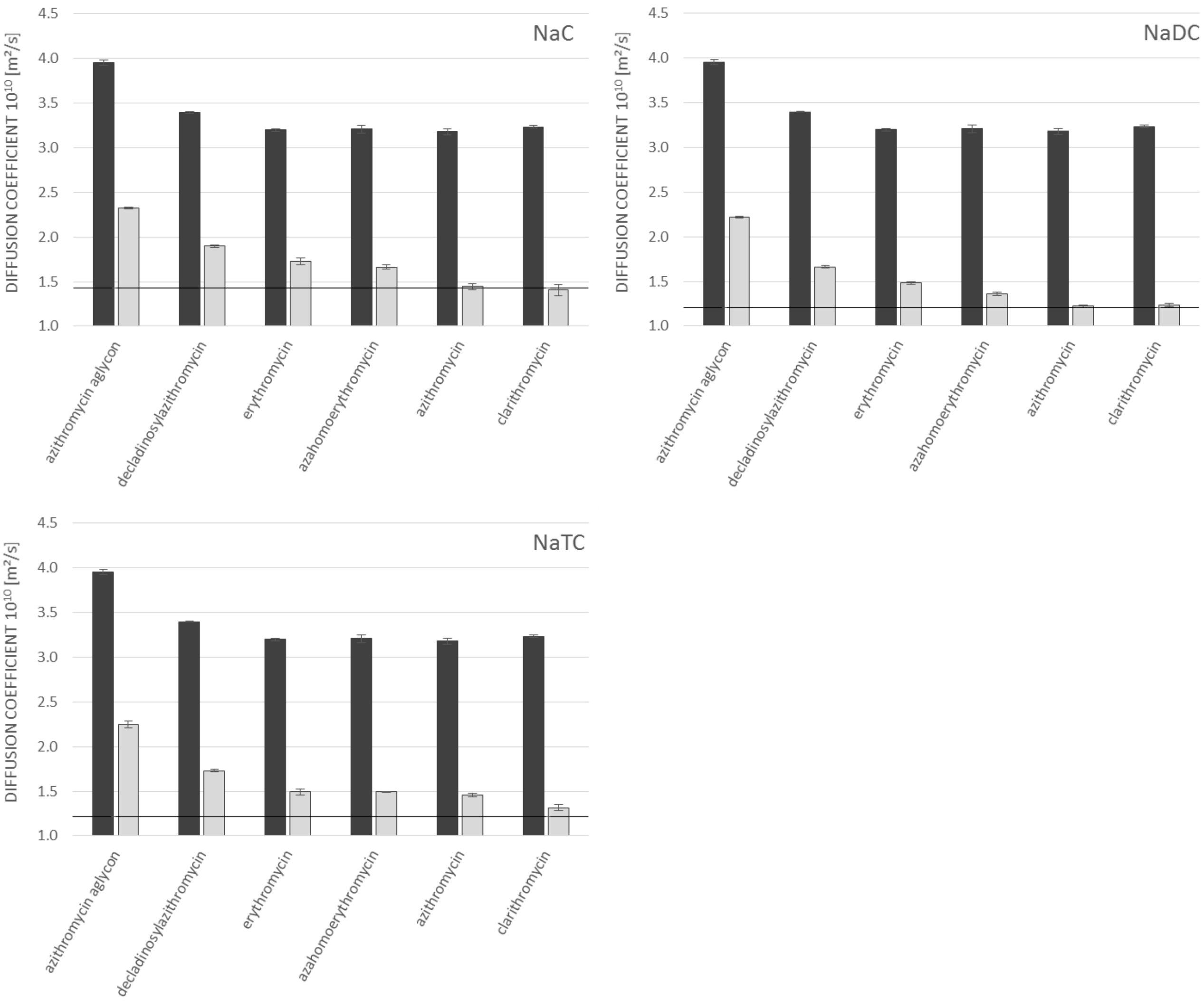


**Supporting Figure 2.** Diffusion coefficients of free antibiotic (black) and in bile salt (grey) in pure $D_2O$. The horizontal line represents the micellar diffusion $D_{mic}$.

**Supporting Figure 3:**

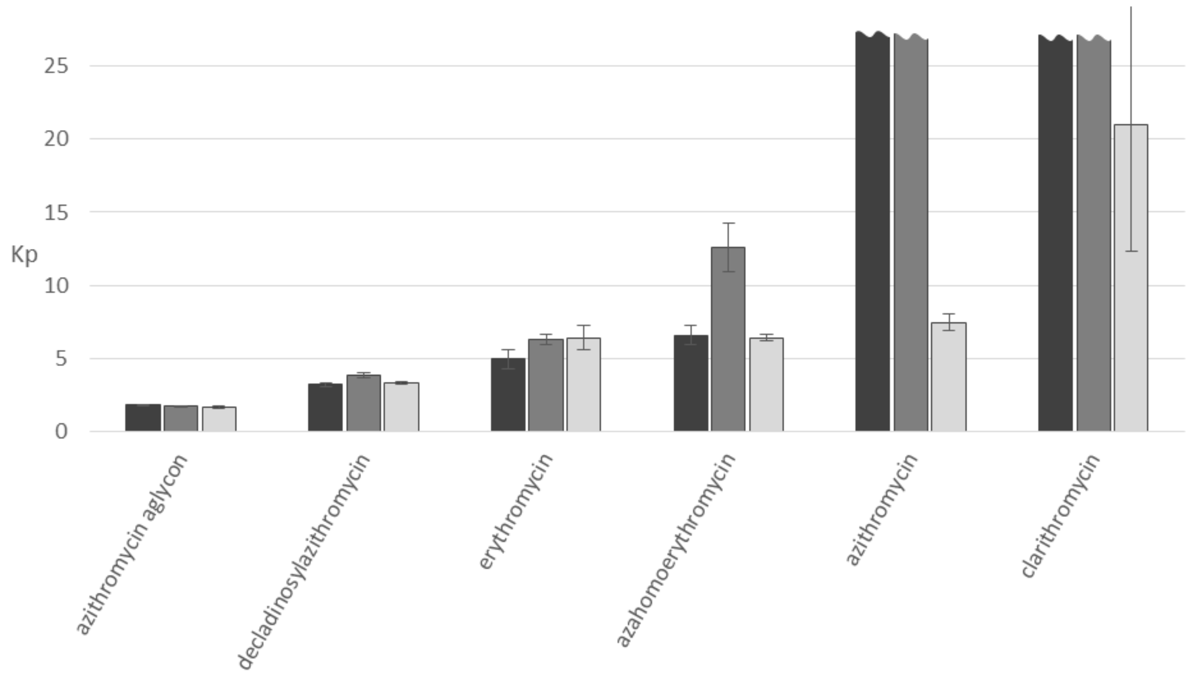


**Supporting Figure 3.** Black: NaC, dark grey: NaDC, light grey: NaTC in pure $D_2O$. Mole fraction partition coefficients beyond ~ 40 are ill-defined and indicated by the wave-shaped ends.

**Supporting Figure 4:**

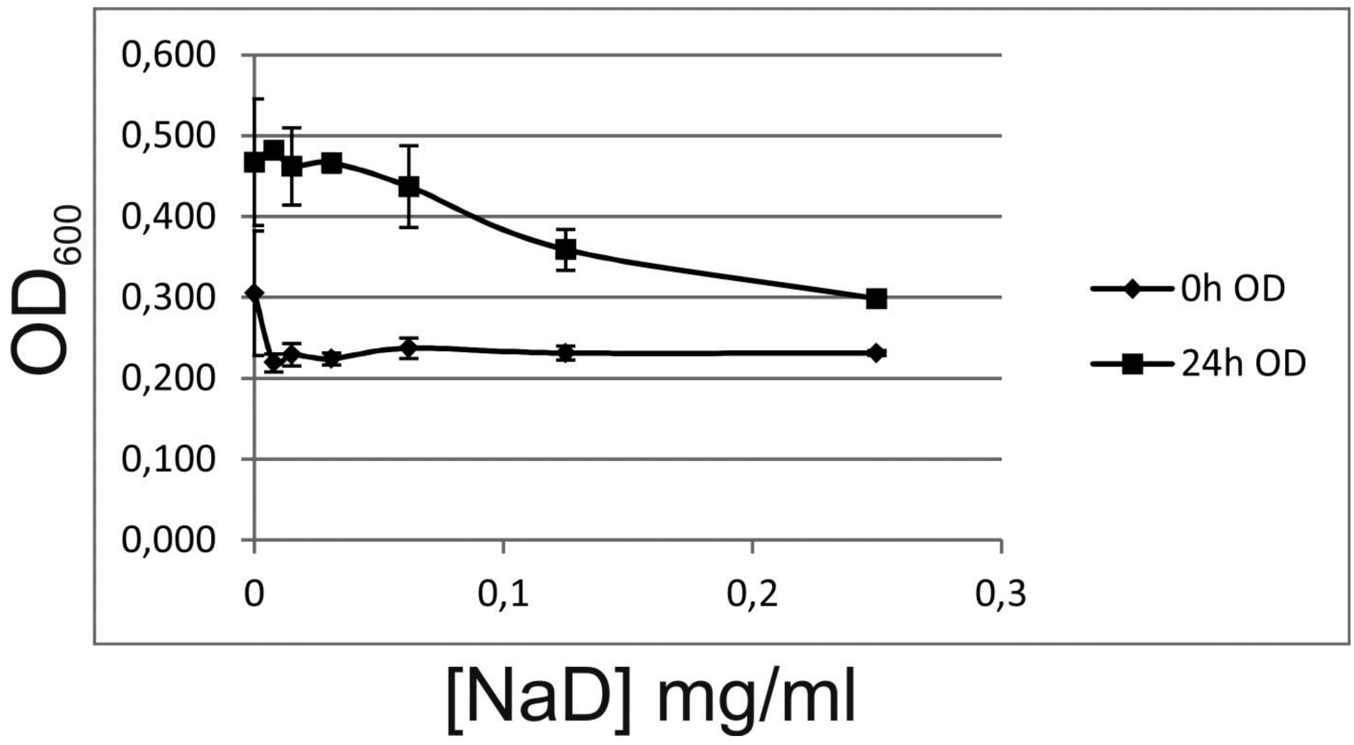


**Supporting Figure 4:**

Optical density (at 600 nm) of ETEC (enterotoxic *E. coli*) cells right after inoculation (0h) and after 24 hours as a function of sodium cholate concentration (in mg NaD per ml).

**Supporting Table 1.** Mole fraction partition coefficients in NaC 100mM, NaDC 50mM and NaTC 50mM in a solvent of pure $D_2O$. *$K_p$ values above ~40 are unreliable due to huge standard deviations.

| | azithromycin aglycone | decladinosyl-azithromycin | erythromycin | azahomo-erythromycin | azithromycin | clarithromycin |
|---|---|---|---|---|---|---|
| NaC | 1.82 +/- 0.04 | 3.22 +/- 0.12 | 4.94 +/- 0.64 | 6.60 +/- 0.63 | > 42.5 * | > 60.9* |
| NaDC | 1.71 +/- 0.03 | 3.83 +/- 0.16 | 6.29 +/- 0.38 | 11.6 +/- 1.66 | > 68.2 * | > 41.2 * |
| NaTC | 1.67 +/- 0.08 | 3.31 +/- 0.12 | 6.40 +/- 0.85 | 6.39 +/- 0.22 | 7.47 +/- 0.53 | 21.0 +/- 8.72 |

## Experimental

*NMR Spectroscopy, using pure $D_2O$ as solvent*

For the interaction studies in $D_2O$ we used saturated solution of the macrolides. They were obtained by adding an excessive amount of the macrolide and sonicating the sample for 20-30 minutes. To get rid of the remaining solid antibiotic, the sample was centrifuged and the liquid phase was used for further experiments.
